# Metabolic syndrome: definitions and controversies

**DOI:** 10.1186/1741-7015-9-48

**Published:** 2011-05-05

**Authors:** Eva Kassi, Panagiota Pervanidou, Gregory Kaltsas, George Chrousos

**Affiliations:** 1 Department of Biochemistry, National and Kapodistrian University of Athens, Athens, Greece; 2 First Department of Paediatrics, National and Kapodistrian University of Athens, Athens, Greece; 3 Department of Pathophysiology, National and Kapodistrian University of Athens, Athens, Greece

## Abstract

Metabolic syndrome (MetS) is a complex disorder defined by a cluster of interconnected factors that increase the risk of cardiovascular atherosclerotic diseases and diabetes mellitus type 2. Currently, several different definitions of MetS exist, causing substantial confusion as to whether they identify the same individuals or represent a surrogate of risk factors. Recently, a number of other factors besides those traditionally used to define MetS that are also linked to the syndrome have been identified. In this review, we critically consider existing definitions and evolving information, and conclude that there is still a need to develop uniform criteria to define MetS, so as to enable comparisons between different studies and to better identify patients at risk. As the application of the MetS model has not been fully validated in children and adolescents as yet, and because of its alarmingly increasing prevalence in this population, we suggest that diagnosis, prevention and treatment in this age group should better focus on established risk factors rather than the diagnosis of MetS.

## Introduction

Metabolic syndrome (MetS) is a complex disorder with high socioeconomic cost that is considered a worldwide epidemic. MetS is defined by a cluster of interconnected factors that directly increase the risk of coronary heart disease (CHD), other forms of cardiovascular atherosclerotic diseases (CVD), and diabetes mellitus type 2 (DMT2). Its main components are dyslipidemia (elevated triglycerides and apolipoprotein B (apoB)-containing lipoproteins, and low high-density lipoproteins (HDL)), elevation of arterial blood pressure (BP) and dysregulated glucose homeostasis, while abdominal obesity and/or insulin resistance (IR) have gained increasing attention as the core manifestations of the syndrome. Recently, other abnormalities such as chronic proinflammatory and prothrombotic states, non-alcoholic fatty liver disease and sleep apnea have been added to the entity of to the syndrome, making its definition even more complex. Besides the many components and clinical implications of MetS, there is still no universally accepted pathogenic mechanism or clearly defined diagnostic criteria. Furthermore, there is still debate as to whether this entity represents a specific syndrome or is a surrogate of combined risk factors that put the individual at particular risk. A main evolving aspect of MetS is its increasing prevalence in both childhood and young adulthood and the future implications to the global health burden this may confer. In the present work we discuss the importance of establishing clear criteria to define MetS, highlighting the latest research, which we use to provide a critical review of currently existing controversies in this field and expand on the childhood and adulthood aspect of the syndrome.

### Currently used criteria to define MetS

Historically, Reaven was the first to put forward the concept of 'syndrome X', (which he later renamed MetS), hypothesizing that it was a central feature in the development of CHD and DMT2, mainly through target tissue resistance to insulin action [1]. Since then, many international organizations and expert groups, such as the World Health Organization (WHO), the European Group for the study of Insulin Resistance (EGIR), the National Cholesterol Education Program Adult Treatment Panel III (NCEP:ATPIII), the American Association of Clinical Endocrinology (AACE), the International Diabetes Federation (IDF), and the American Heart Association/National Heart, Lung, and Blood Institute (AHA/NHLBI), have attempted to incorporate all the different parameters used to define MetS (Appendix 1).

The first attempt was made in 1998 by the WHO, which proposed that MetS may be defined by the presence of IR or its surrogates, impaired glucose tolerance (IGT) or DMT2, as essential components of the syndrome, along with at least two of the following parameters: raised BP, hypertriglyceridemia and/or low HDL-cholesterol, obesity (as measured by waist/hip ratio or body mass index (BMI)), and microalbuminuria [2]. Shortly thereafter, the EGIR excluded microalbuminuria as an integral component of the syndrome, while it required hyperinsulinemia to be present [3]. In addition, waist circumference and not BMI was regarded as the main indicator to assess obesity, while introducing different cut-offs from those previously used for the other components of the syndrome. In 2001, the NCEP:ATPIII published a new set of criteria that included waist circumference, blood lipids, BP, and fasting glucose [4]. The NCEP:ATPIII definition differed from both the WHO and EGIR definitions in that IR was not considered as a necessary diagnostic component. In 2005, the International Diabetes Federation (IDF) published newer criteria in an attempt to define the syndrome more precisely so that it could be used by different clinical and research groups. The aim of this new definition was to enable comparisons between study results, in the hope that it would be a better predictor of risk particularly for CHD, stroke and DMT2 [5]. The IDF introduced abdominal obesity as a prerequisite of the diagnosis of MetS, with particular emphasis on waist measurement as a simple screening tool that was also adopted by AHA/NHLBI [6].

The remaining four components of MetS were identical in the AHA/NHLBI definition to those of the IDF, although abdominal obesity was defined differently. The IDF recommended that the threshold for waist circumference in Europeans should be 94 cm for men and 80 cm for women, while the AHA/NHLBI recommended cut-off points of 102 and 88 cm, respectively.

Currently, the two most widely used definitions are those of the NCEP:ATP III and IDF focusing specifically on waist circumference, which is a surrogate measure of central obesity. In contrast, the AACE, WHO and the EGIR definitions are all largely focused on insulin resistance.

However, a major problem with the WHO and NCEP:ATPIII definitions has been their applicability to different ethnic groups, especially when trying to define obesity cut-offs. This is particularly evident for the risk of DMT2, which is apparent at much lower levels of obesity in Asians compared to Europeans. The IDF, having recognized the difficulties in identifying unified criteria for MetS that were applicable across all ethnicities, has proposed a new set of criteria with ethnic/racial specific cut-offs [7]. However, even in Westerners, a difference of 14 cm in current abdominal obesity criteria across genders may be debatable, leading to dilution of MetS in women or a failure of encompassing men with MetS at increased cardiometabolic risk.

In view of these difficulties and until more evidence that can elucidate the cause of MetS accumulate, the Joint Interim Statement (consensus definition in Appendix 1) highlighting that there should be no obligatory component for MetS but rather all individual components should be considered important on risk prediction, is currently mostly accepted.

Although prevalence estimates for the syndrome have been mostly similar in any given population regardless of the definition used, different individuals are identified [8]. This is attributed to the different focus of each definition, from the glucocentric WHO definition to an obesity-centric IDF one, and to a collection of statistically related CVD risk factors by the NCEP:ATPIII definition. Taking into account the burgeoning epidemic of DMT2 and CVD worldwide, the need for one practical definition that would identify accurately individuals with MetS is becoming imperative.

### Epidemiology of MetS according to the various definitions used

Clearly, the prevalence of MetS varies and depends on the criteria used in different definitions, as well as the composition (sex, age, race and ethnicity) of the population studied [9]. No matter which criteria are used, the prevalence of MetS is high and rising in all western societies, probably as a result of the obesity epidemic [10-12]. According to National Health and Examination Survey (NHANES) 2003-2006 [13], approximately 34% of people studied met the NCEP:ATPIII revised criteria for MetS. Differences in the age-adjusted prevalence estimates using the various definitions of MetS within three NHANES cohorts (1988-1994, 1999-2002 and 2003-2006) are shown in Table 1. Using the revised NCEP:ATPIII criteria, the estimated prevalence of MetS increased up to 5% during the last 15 years. The WHO criteria, although more restrictive, estimated nearly the same prevalence of MetS, whereas the IDF definition which adopted a lower cut-off point for waist circumference, estimated a higher prevalence [14]. Despite the differing prevalence estimates when employing the various definitions, they all add up to a shocking figure of a vast proportion of the population being at high risk of developing CHD and DMT2.

**Table 1:** Age-adjusted prevalence according to MetS definition within NHANES cohorts.

	N	ATPIII 2001	ATPIII revised	WHO	IDF
NHANES 1988-1994 [143]	8,814	23.7%			

NHANES 1988-1994 [144]	8,608	23.9%		25.1%	

NHANES 1988-1994 [145]	6,436	24.1%	29.2%		

NHANES 1999-2002 [145]	1,677	27.0%	32.3%		

NHANES 1999-2002 [10]	3,601		34.6%		39.1%

NHANES 2003-2006 [13]	3,423		34%		

ATPIII = National Cholesterol Education Program Adult Treatment Panel III; IDF = International Diabetes Federation; MetS = metabolic syndrome; NHANES = National Health and Examination Survey; WHO = World Health Organization.

Both the unadjusted prevalence and age-adjusted prevalence of MetS increased from NHANES-III (1988-1994) to NHANES 1999-2006, from 27.9% to 34.1% and 29% to 34.2%, respectively, but remained the same over the last NHANES cohorts (1999-2002, and 2003-2006 cohort). In countries from areas other than Europe and Africa, the IDF guidelines also identify a greater prevalence of MetS than the NCEP:ATPIII [15-19]. A similar prevalence of MetS in the Iranian population was recently reported, applying both the IDF and ATPIII criteria (32.1% and 33.2% respectively) [20].

In the NHANES 2003-2006 cohort, the prevalence of MetS was found to increase with age: approximately 20% of males and 16% of females under 40 years of age, 41% of males and 37% of females between 40-59 years, and 52% of males and 54% of females 60 years and over [13]. The trend for a higher prevalence of MetS with advancing age was similar to that observed in other populations [8,21-25]. This increase with age continues up to the sixth decade; however, different studies have estimated a variable prevalence after the sixth or seventh decades, probably as individuals most susceptible to obesity-related mortality have already died [22,24,26].

The prevalence of MetS increases even more dramatically as BMI increases. In the NHANES 2003-2006 cohort, overweight males and females were found, respectively, to be more than 6 and 5.5 times as likely to meet the criteria for MetS compared to underweight and normal weight individuals. In obese males and females compared to underweight and normal weight individuals these figures spiked to 32 and 17 times, respectively [13]. Similarly to western societies, the prevalence of MetS is rapidly increasing in developing countries, ranging from 9.8% in male urban North Indians to 42% in female urban Iranians [27]. This increase is observed regardless of the criteria used and reflects the transition from a traditional to a Western-like lifestyle. The emergence of obesity and MetS in developing countries is related to a number of factors. Demographic transition (shift to low fertility, low mortality, and higher life expectancy), and epidemiological transition (from widely prevalent infectious diseases to a pattern of a high prevalence of lifestyle related diseases) evolved in developing countries as they become economically more resourceful, leading to significant shifts in dietary and physical activity patterns. These changes cause significant effects on body composition and metabolism, often resulting in an increase in BMI, generalized and abdominal obesity, and an increase in dyslipidemia and DMT2 [27].

It should be noted however, that even lean individuals may develop features of MetS adding further to the complexity of its pathogenesis [28].

Thus, the importance of identifying markers for MetS to supplement age-related and obesity-related measures cannot be overstated. Understanding how to use definition criteria in clinical settings will aid physicians in treating the right cohort of at-risk patients.

### Morbidities related to MetS

The need to precisely define MetS stems from the need to detect accurately individuals at high risk for CVD and DMT2. All the components of the various MetS definitions are involved in conferring risk for CVD and DMT2. In particular, the three components of atherogenic dyslipidemia (increased low-density lipoprotein (LDL), decreased HDL and high blood triglyceride concentrations) are individually associated with a cardiovascular risk [29], while IR significantly increases the risk of developing DMT2, although approximately 25% of insulin resistant patients have normal glucose tolerance [30]. Central obesity has been shown in several studies to be associated with an increased risk of CVD and DMT2 [31].

Several epidemiological studies have confirmed the increased risk of CVD in individuals with MetS, independently of the diagnostic criteria used [32-39]. Overall a range of 1.5-3 times greater risk of CVD and CHD mortality has been found in several prospective studies (REFS), whereas a recent meta-analysis showed that MetS was associated with a twofold increase in cardiovascular outcomes and a 1.5-fold increase in all-cause mortality [40]. It should be noted however, that several studies amongst which the Casale Monferrato Study and PROSPER conducted in older people, failed to reveal an association between MetS and an increased risk of CVD [41,42]. Due to these inconsistencies several recent studies have aimed to investigate which of the proposed definitions of MetS is particularly related to excessive CVD risk, and thus which one should be implemented in clinical practice. A recent meta-analysis suggested that the WHO definition was associated with a slightly greater risk than the NCEP:ATPIII definition [43]. It is of interest that the INTERHEART study, the first large-scale multiethnic international investigation, has demonstrated that using either the WHO or IDF definition, the presence of MetS is associated with a > 2.5-fold increase in the risk of acute myocardial infarction (MI) [44], while Cabre *et al*. recently reported similar findings for CVD using these definitions [45].

The assessment of whether the risk of MetS on MI is greater than the risk conferred by the sum of individual component risk factors has also been studied. A recent report suggested that the MetS-related CVD risk was more than that of the sum of its parts in subjects with MetS, with or without DMT2 [46]. In the recent INTERHEART study, MI risk increased as more component factors of MetS were present, and this is consistent with previous observations showing that the presence of more risk factors was associated with an incremental increase in subclinical atherosclerosis and incident CHD [38,47-49]. One major point of note was that the risk of CHD when three components of MetS were present did not appear to be greater than the risk of individual components such as DMT2 and hypertension, and this was in agreement with other reports [50,51]. In this regard, in a recent appraisal of MetS, the American Diabetes Association (ADA) in conjunction with the European Association for the Study of Diabetes, issued a joint statement raising concerns over the value of using MetS as a CVD risk marker and recommended that clinicians should evaluate and treat all CVD risk factors without considering whether a patient meets the criteria for diagnosis of MetS [52].

The observation of the presence of MetS predicting the risk for DMT2 has also been examined by numerous studies; according to these reports, it is well accepted that the presence of MetS not only increases this risk, but is also highly predictive of new-onset DMT2 [6,42,53-55]. Indeed, MetS is associated with an approximately five times higher risk for incident DMT2 [56].

The presence of MetS predicting the incidence of DMT2 also varies depending on how MetS is defined. It has previously been demonstrated that definitions like that of the WHO, which imposes as a prerequisite the presence of impaired fasting glucose (IFG) and/or IGT, confer a higher risk than the NCEP:ATPIII and IDF definitions, which consider elevated fasting plasma glucose as an essential, but not required component [57]. IFG and IGT can predict the development of DMT2, independently of other components of MetS [56]. However, recent studies have shown that the IDF and NCEP:ATPIII definitions of MetS predicted DMT2 at least as well as the WHO definition [58,59].

Although the presence of MetS can predict the CVD and DMT2 risk, it cannot estimate the exact risk, as a significant part may be related to other factors such as age, smoking or gender. In particular, women have higher concentrations of inflammatory markers, such as high sensitivity C reactive protein (hs-CRP), compared to men, attributed possibly to their increased accumulation of subcutaneous and/or visceral fat [60,61]. Additionally, factors others than those included in the existing definitions of MetS, such as endothelial dysfunction, small dense oxidized LDL, insulin resistance, prothrombotic tendency and a proinflammatory state that are essential components and determinant of future cardiometabolic risk have been left out. Indeed, a substantial amount of knowledge on cardiometabolic risk is provided by markers that define a proinflammatory state, such as hs-CRP, γ-glutamyltransferase (γ-GT), uric acid, apoB, apoE, fibrinogen, along with the associated dysfunction of apolipoprotein A-I (ApoA-I) and HDL [62-67]. In particular, HDL dysfunctionality is closely linked to obesity and low-grade inflammation yet seems to act partly independently of them. Although it is not as yet clear what is the exact contribution of these risk factors to the development of CVD and DMT2, it is probable that along with the mechanisms delineated further below, may account for the residual risk not attributed to the traditional risk factors of these disorders.

### Conditions contributing to the pathogenesis of MetS (Figure 1)

**Figure 1:**
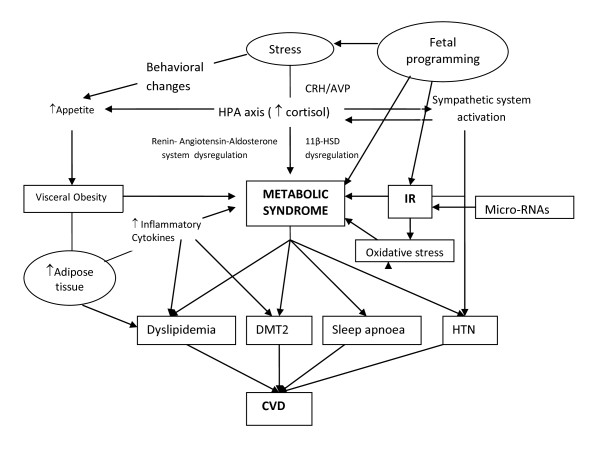
**A schematic image of the conditions  implicated in the pathophysiology of the metabolic syndrome and their potential interactions**. IR: Insulin Resistance; HTN: Hypertension; HPA axis : Hypothalamic-Pituitary-Adrenal Axis; DMT2: Diabetes Mellitus type 2; CVD: Cardiovascular disease; CRH: Corticotropin Releasing Hormone; AVP: Arginine Vasopressin.

Despite advances in pathophysiology and delineation of risk factors that predispose to MetS, there are many key aspects that remain unclear. The great variation in susceptibility and age of onset in individuals with a very similar risk profile, suggests a major interaction between genetic and environmental factors [68]. Although obesity and IR remain at the core of the pathophysiology of MetS, a number of other factors such as chronic stress and dysregulation of the hypothalamic-pituitary-adrenal (HPA) axis and autonomic nervous system (ANS), increases in cellular oxidative stress, renin-angiotensin-aldosterone system activity, and intrinsic tissue glucocorticoid actions, as well as currently discovered molecules such as micro RNAs can also be involved in its pathogenesis (Figure 1).

#### Conditions epidemiologically confirmed

##### Obesity/insulin resistance and MetS

Although not all overweight or obese individuals are metabolically disturbed, the majority are IR [69-71]. Central obesity is thought to be an early step, as visceral adipose tissue secretes a variety of bioactive substances termed adipocytokines, such as leptin, resistin, tumor necrosis factor α (TNFα), interleukin-6 (IL-6), and angiotensin II which induce IR, along with plasminogen activator inhibitor 1 (PAI-1), which is related to thrombogenic vascular diseases [72]. Notably, adiponectin, an important adipocytokine that protects against the development of DMT2, hypertension, inflammation, and atherosclerotic vascular diseases, is decreased in individuals with visceral fat accumulation, and this may be causally related to MetS [73,74]. Moreover, newly recognized adipocytokines such as visfatin, as well as enzymes expressed in adipose tissue, such as neprilysin, and growth factors, like fibroblast growth factor 21, an important regulator of glucose and lipid metabolism, are currently under investigation regarding their role in the pathogenesis of MetS [75-77]. In particular, visfatin has been suggested to exert insulin-mimicking/sensitizing effects but can also contribute to the inflammatory processes by triggering cytokine production and nuclear factor κB (NF-κB) activation, while *vice versa *visfatin secretion is upregulated in response to proinflammatory cytokines [78].

Other compounds produced by adipose tissue possibly implicated in the pathogenesis of MetS, are the non-esterified free fatty acids (FFAs). In the presence of IR the process of FFAs mobilization from stored adipose tissue triglycerides is accelerated. In the liver, FFAs result (due to hepatic insulin resistance) in increased production of glucose and triglycerides and secretion of very low-density lipoprotein (VLDL), maintaining a vicious cycle. FFAs also reduce insulin sensitivity in muscle by inhibiting insulin-mediated glucose uptake and increase fibrinogen and PAI-1 production [9,79].

#### Conditions related to MetS without epidemiological confirmation

##### Chronic stress: dysregulation of HPA axis/ANS and MetS

Chronic hypersecretion of stress mediators, such as cortisol, in individuals with a genetic predisposition exposed to a permissive environment, may lead to visceral fat accumulation as a result of chronic hypercortisolism, low growth hormone secretion and hypogonadism [80,81]. Moreover, hypercortisolism directly causes IR of peripheral target tissues in proportion to glucocorticoid (GC) levels and a particular target tissue's sensitivity to them as shown by studying polymorphisms of the glucocorticoid receptor gene [82]. These hormonal alterations may lead to reactive insulin hypersecretion, and increasing visceral obesity and sarcopenia, resulting to dyslipidemia, hypertension and DMT2 [83]. Stress-related IL-6 hypersecretion plus adipose-tissue-generated inflammatory hypercytokinemia, as well as hypercortisolism, contribute to increased production of acute phase reactants and blood hypercoagulation, which have been recently recognized as components of MetS [84,85].

Moreover, since intracellular GC levels are regulated by 11β-hydroxysteroid dehydrogenase type 1 (11β-HSD1), which converts inactive cortisone to cortisol, a large number of studies have focused on evaluating tissue specific alterations in 11β-HSD1 expression and activity in obesity and IR. In obesity global 11β-HSD1 activity, as measured by urinary corticosteroid metabolite analysis, is impaired [86,87], while selective 11β-HSD1 inhibitors, are in development with promising results showing improvements in metabolic profile in rodents [88].

A possible association between visceral fat/insulin resistance, inflammatory cytokines, stress hormones, and sleep apnea has recently been suggested [89-91]. Visceral obesity/insulin resistance, determined by both genetic/constitutional and environmental factors, may be the principal culprit leading to sleep apnea, which, in turn, may accelerate these metabolic abnormalities, possibly through progressive elevation of stress hormones and cytokines such as noradrenaline, cortisol, IL-6, and TNFα [92].

Apart from the stress HPA axis and its end effectors glucocorticoids, another system, the circadian CLOCK system may also be implicated in the pathogenesis of MetS. Interestingly, most of the metabolic phenotypes associated with dysregulation of the CLOCK system and the HPA axis overlap [93].

##### Cellular oxidative stress/renin-angiotensin-aldosterone system and MetS

Emerging evidence suggests that nitric oxide (NO), inflammatory and oxidative stress also play important roles in the pathophysiology of MetS hypertension and DMT2 [94,95]. Increased production of reactive oxygen species (ROS) in numerous tissues, including skeletal muscle and cardiovascular tissues, has been linked (amongst others) to activation of the renin-angiotensin-aldosterone system [96,97], which is also implicated in the development of IR [98]. Elevation of angiotensin II can induce IR via reactive oxygen species (ROS) production in various tissues, including vascular smooth muscle and skeletal muscle in patients with MetS [96,98]. Furthermore, either use of angiotensin II type 1 receptor antagonists or genetic knockout of angiotensin II type 1 receptor are known to effectively attenuate lipid accumulation in the liver [99-105].

##### Micro RNAs and MetS

Micro RNAs (miRNAs) play important regulatory roles in a variety of biological processes including adipocyte differentiation, metabolic integration, IR and appetite regulation [106]. Although the exact mechanism of action remains to be elucidated, miRNAs may regulate cellular gene expression at the transcriptional or post-transcriptional level, by suppressing translation of protein-coding genes, or cleaving target messenger RNAs (mRNAs) to induce their degradation, through imperfect pairing with target mRNAs [107,108]. Antagomirs (cholesterol conjugated antisense oligonucleotides) which target and silence miRNAs, as evidenced by hepatic *miR-122 *blockade *in vivo *[109], have already been successfully tested in a phase I clinical trial. Further studies are needed to explore the full potential of miRNAs as novel biomarkers and therapeutic agents against MetS.

##### Fetal/developmental basis of MetS

The concept of fetal/developmental origin of MetS, since the first study linking intrauterine undernutrition with later obesity, continues to raise interest [110]. Evidence from both human [111,112] and animal studies [113,114] suggests that the nutritional, hormonal, and metabolic environment of the mother, as well as the early postnatal environment, may permanently reprogram the structure and physiology of the offspring toward the development of metabolic disease, in later life [80,84,115,116].

Since *in vitro *fertilization (IVF) has been widely used, the possible effect of IVF (as a result of either intrauterine growth restriction or periconceptual manipulation of the blastocysts *per se*) in the incidence of MetS manifestations has been studied, producing conflicting results [117-119]. More prospective studies on the metabolic profile of children conceived by IVF, with longer follow-up are necessary to draw safe conclusions.

Although the role of the all these components as integral parts of MetS has not been evaluated in epidemiological and interventional studies, they may represent the missing link that provides full susceptibility to CVD besides the traditionally accepted components of MetS. This is particularly relevant for the fetal programming as it may suggest intervention at an earlier stage with lifestyle therapies, since the incidence of MetS in children and adolescents is increasing alarmingly.

### Is the pediatric metabolic syndrome real or a myth? A developmental perspective

The increasing worldwide prevalence of childhood obesity and, in parallel, of DMT2 in the young [120,121], has highlighted the importance of MetS diagnosis in children and adolescents, as a state of high risk for progression to later disease. Since the first publication of MetS in children, in 1999, [122], a growing interest emerged investigating MetS prevalence and the potential utility of this diagnosis, as well as therapeutic interventions in adolescents fulfilling it. Later, findings from the Third National Health and Nutrition Examination Survey (NHANES), 1988-1994 [123] revealed that 4.2% of adolescents in general, and almost 30% of overweight and obese adolescents, in the US met diagnostic criteria for MetS. A variety of subsequent studies [120,123-131], using three or four criteria and variable definitions, revealed diversity in MetS prevalence in childhood. The NHANES 1999-2000, using the ATPIII definition modified for age, identified a further increase in the prevalence of MetS among US adolescents, from 4.2% in NHANES III (1988-1994) to 6.4% in NHANES (1999-2000) [127]. The prevalence of MetS was almost exclusively found to be high among obese adolescents.

#### Definitions

Similarly to adults, no general consensus exists regarding the definition of MetS in children and adolescents [132]. Furthermore, studies published so far have used their own set of variables, number of criteria (three or four) and different cut-off points to define risk factors associated with MetS. Obesity has been defined as the 85th to 97th percentile of BMI or waist circumference, while accordingly, a variety of cut-off percentiles have been used for blood pressure, triglycerides, HDL, insulin and glucose [133]. In 2007, a consensus report was published by the IDF group [134], including three age groups: 6 to < 10, 10 to < 16 and 16 + years (adult criteria). Based on this report, obesity is defined as waist circumference ≥ 90th percentile, or adult cut-off if lower, while all other parameters are defined based on absolute numbers, rather than percentiles. These numbers are: ≥ 150 mg/dl for triglycerides (or specific treatment for triglycerides), < 40 mg/dl for HDL and < 50 mg/dl in females older than 10 (or specific treatment for HDL), ≥ 130 mmHg for systolic and ≥ 85 mmHg for diastolic blood pressure (or treatment of previously diagnosed hypertension) and fasting plasma glucose ≥ 100 mg/dl or known type 2 diabetes. The rationale of using absolute numbers as cut-offs is based on the heterogeneity of clinical, biochemical and hormonal values during childhood and adolescence, as well as, on the large diversity of proposed percentile cut-offs of different definitions. The IDF definition is presented in detail in Table 2.

**Table 2:** International Diabetes Federation (IDF) definition for pediatric metabolic syndrome (MetS) [134]

Age group (years)	Obesity (WC)	Triglycerides	HDL-C	Blood pressure	Glucose
6 to < 10^a^	≥ 90th percentile				

10 to < 16	≥ 90th percentile or adult cut-off if lower	≥ 1.7 mmol/l (≥ 150 mg/dl)	< 1.03 mmol/l (< 40 mg/dl)	Systolic BP ≥ 130 or diastolic BP ≥ 85 mm Hg	FPG ≥ 5.6 mmol/l (100 mg/dl)^b ^or known T2DM

16+ (adult criteria)	WC ≥ 94 cm for Europid males and ≥ 80 cm for Europid females, with ethnic-specific values for other groups^c^	≥ 1.7 mmol/l (≥ 150 mg/dl) or specific treatment for high triglycerides	< 1.03 mmol/l (< 40 mg/dl) in males and < 1.29 mmol/l (< 50mg/dl) in females, or specific treatment for low HDL	Systolic BP ≥ 130 or diastolic BP ≥ 85 mm Hg or treatment of previously diagnosed hypertension	FPG ≥ 5.6 mmol/l (100 mg/dl)^b ^or known T2DM

Diagnosing metabolic syndrome requires the presence of central obesity plus any two of the other four factors.

^a^Metabolic syndrome cannot be diagnosed, but further measurements should be made if there is a family history of metabolic syndrome, T2DM, dyslipidemia, cardiovascular disease, hypertension and/or obesity.

^b^For clinical purposes, but not for diagnosing MetS, if FPG: 5.6-6.9 mmol/l (100-125 mg/dl) and not known to have diabetes, an oral glucose tolerance test should be performed.

^c^For those of South and South-East Asian, Japanese, and ethnic South and Central American origin, the cut-offs should be ≥ 90 cm for men, and ≥ 80 cm for women. The IDF Consensus group recognizes that there are ethnic, gender and age differences, but research is still needed on outcomes to establish risk.

BP = blood pressure; FPG = fasting plasma glucose; HDL-C = high-density lipoprotein cholesterol; IDF = International Diabetes Federation; T2DM = type 2 diabetes mellitus; WC = waist circumference.

#### The pediatric perspective: challenges in conceptual models

The main concern for pediatric clinicians is that all childhood MetS definitions originate from adult definitions and use criteria extrapolated from an adult diagnosis to a younger age group, while, in fact, the utility and predictive value of this diagnosis in young age groups has not been fully established. Indeed, large longitudinal studies linking pediatric MetS with adult cardiovascular disease are limited, and although it is hypothesized that MetS in childhood is related to MetS in adulthood, this hypothesis has not yet been tested. A second important issue is the lack of a developmental perspective in MetS definition: MetS as an entity is developing progressively, according to age and pubertal changes, so that the full MetS cannot in general be easily diagnosed in childhood [124]. Developmentally-appropriate risk or protective factors, such as gestational age, birth weight and breastfeeding, as well as parental obesity and family history, are not typically taken into account [133]. More importantly, none of the MetS definitions consider the influences of growth and puberty, for instance the 'normal' insulin resistance in puberty [135,136], the changes in fat and fat-free mass and the changes in growth and sex steroid secretion. Further to these changes, it has been shown that in obese children, insulin resistance (as measured by the homeostasis model assessment-insulin resistance (HOMA-IR) index) increases progressively across Tanner stages and is higher in all pubertal stages than in normal weight children [137].

#### MetS consistency in adolescence

Recently published studies examined MetS stability in large epidemiological samples of adolescents, using factor analysis. It was found that, though metabolic risk factor clustering was consistent, the categorical diagnosis of MetS was not stable during adolescence [138], including both gain and loss of diagnosis. A second, more recent study from the same group, examined the stability of three alternative models of MetS factor structure across three developmental changes [139]. The researchers suggested that the concepts used to support the utility of MetS in the young do not fit to pediatric populations and may vary by maturation. In addition to these large epidemiological studies, from US, clinical data also support this idea. Our research group examined the prevalence and stability of MetS diagnosis in children and adolescents aged 8-16. Data from our cohort showed that in a clinical population of obese children followed at our obesity outpatient clinic, almost 10% had the full MetS, and another 30% had partial MetS (two positive parameters of MetS). When examining the effects of puberty, we found that pubertal children had a higher prevalence of full and partial MetS than the prepubertal population. However, this diagnosis presented a within-person variability when examined at different time points during adolescence [140]. This new information is further supported by recently published data from the Bogalusa Heart Study and the Cardiovascular Risk in Young Finns [141]. This study has shown that although children and adolescents with MetS are indeed at an increased risk of adult MetS, subclinical atherosclerosis and diabetes type 2, the BMI alone is an equally accurate measure as MetS in identifying youth at risk for adult MetS and subsequent atherosclerotic disease.

#### Clinical implications for pediatric MetS

MetS as a concept was originally developed to identify adults at a greatest risk for CVD and DMT2, however, the application of this model has not yet been fully validated in children and adolescents. Furthermore, the effects of growth and puberty on reference values is a critical issue, because diverse age-dependent cut-off points are needed to define a pathological state, such as MetS. Longitudinally, the high level of diagnostic inconsistency through adolescence suggests that MetS classification may not be a valuable method for risk identification in the pediatric age group.

These limitations, as well as new data from longitudinal studies in pediatric MetS, suggest that prevention and treatment in childhood and adolescence should better focus on established risk factors rather than the diagnosis of MetS. Pediatric clinicians may put more emphasis on healthy lifestyle promotion and obesity prevention and treatment rather than targeting specific metabolic alterations. Indeed, advocating weight maintenance rather than weight loss during the years of physical growth, may lead to BMI reduction and cardiovascular risk minimization and may be a more cost-effective approach than pursuing fluctuating biochemical abnormalities.

## Conclusions

Due to its impact upon health and financial implications, the mechanisms that contribute to the pathogenesis of MetS remain under intense investigation since their understanding may help design novel therapeutic strategies. A number of potential mechanisms contributing to the pathogenesis of MetS include fetal programming, dyshomeostasis of the stress system, and the development of a proinflammatory and prothrombotic state as a result of cytokine production and/or dysregulation from the excessive adipose tissue. Delineation of the role of these factors along with the established ones and others that are currently being studied may help clarify the exact pathogenesis of the syndrome and may expand the clinical criteria of MetS. This is particularly important as there is still a need to develop uniform criteria that can be used by different clinical and research groups, enabling comparisons between study results, in the hope to better predictor the risk, for CVD and DMT2. In this direction, further studies exploring the relation of waist circumference thresholds to metabolic risk and cardiovascular outcomes in different populations are encouraged. However, until this aim is achieved, the consensus definition incorporating IDF and AHA/NHLBI is, in our opinion, the most suitable for practical use in clinical medicine. Adoption of these criteria seem to incorporate the most important aspects of the syndrome, recognizing that the risk associated with a particular waist measurement will differ in different populations, albeit with the limitations that it has when applied to mixed ethnicities. Finally, the application of the MetS model has not been fully validated in children and adolescents as yet, suggesting that prevention and treatment in childhood and adolescence should better focus on established risk factors rather than the diagnosis of MetS.

## Appendix 1

### Criteria for metabolic syndrome (MetS) definitions in adults

#### World Health Organization criteria (1998) [2]

Insulin resistance is defined as type 2 diabetes mellitus (DM) or impaired fasting glucose (IFG) (> 100 mg/dl) or impaired glucose tolerance (IGT), plus two of the following:

• Abdominal obesity (waist-to-hip ratio > 0.9 in men or > 0.85 in women, or body mass index (BMI) > 30 kg/m^2^.

• Triglycerides 150 mg/dl or greater, and/or high-density lipoprotein (HDL)-cholesterol < 40 mg/dl in men and < 50 mg/dl in women.

• Blood pressure (BP) 140/90 mmHg or greater.

• Microalbuminuria (urinary albumin secretion rate 20 μg/min or greater, or albumin-to-creatinine ratio 30 mg/g or greater).

### European Group for the Study of Insulin Resistance criteria (1999) [3]

Insulin resistance defined as insulin levels > 75th percentile of non-diabetic patients, plus two of the following:

• Waist circumference 94 cm or greater in men, 80 cm or greater in women.

• Triglycerides 150 mg/dl or greater and/or HDL-cholesterol < 39 mg/dl in men or women.

• BP 140/90 mmHg or greater or taking antihypertensive drugs.

• Fasting glucose 110 mg/dl or greater.

### National Cholesterol Education Program Adult Treatment Panel III (NCEP:ATPIII) criteria (2001) [4]

Any three or more of the following:

• Waist circumference > 102 cm in men, > 88 cm in women.

• Triglycerides 150 mg/dl or greater.

• HDL-cholesterol < 40 mg/dl in men and < 50 mg/dl in women.

• BP 130/85 mmHg or greater.

• Fasting glucose 110 mg/dl* or greater.

* In 2003, the American Diabetes Association (ADA) changed the criteria for IFG tolerance from 110 mg/dl to 100 mg/dl.

### American Association of Clinical Endocrinology criteria (2003) [142]

IGT plus two or more of the following:

• BMI 25 kg/m^2 ^or greater.

• Triglycerides 150 mg/dl or greater and/or HDL-cholesterol < 40 mg/dl in men and < 50 mg/dl in women.

• BP 130/85 mmHg or greater.

### International Diabetes Federation (IDF) criteria (2005) [5]

Central obesity (defined as waist circumference but can be assumed if BMI > 30 kg/m^2^) with ethnicity-specific values,* plus two of the following:

• Triglycerides 150 mg/dl or greater.

• HDL-cholesterol < 40 mg/dl in men and < 50 mg/dl in women.

• BP 130/85 mmHg or greater.

• Fasting glucose 100 mg/dl or greater.

*To meet the criteria, waist circumference must be: for Europeans, > 94 cm in men and > 80 cm in women; and for South Asians, Chinese, and Japanese, > 90 cm in men and > 80 cm in women. For ethnic South and Central Americans, South Asian data are used, and for sub-Saharan Africans and Eastern Mediterranean and Middle East (Arab) populations, European data are used.

### American Heart Association/National Heart, Lung, and Blood Institute (AHA/NHLBI) criteria (2004) [6]

Any three of the following:

• Waist circumference 102 cm or greater in men, 88 cm or greater in women.

• Triglycerides 150 mg/dl or greater.

• HDL-cholesterol < 40 mg/dl in men and < 50 mg/dl in women.

• BP 130/85 mmHg or greater.

• Fasting glucose 100 mg/dl or greater.

### Consensus definition (incorporating IDF and AHA/NHLBI definitions) [7]

Any three of the following:

• Elevated waist circumference (according to population and country-specific definitions).

• Triglycerides 150 mg/dl or greater.

• HDL-cholesterol < 40 mg/dl in men and < 50 mg/dl in women.

• BP 130/85 mmHg or greater.

• Fasting glucose 100 mg/dl or greater.

## Competing interests

The authors declare that they have no competing interests.

## Authors' contributions

EK and GK carried out the literature search, the analysis of the data and wrote the manuscript. NP wrote the pediatric metabolic syndrome section of the manuscript. GC reviewed the manuscript critically. All authors read and approved the final manuscript.

## Pre-publication history

The pre-publication history for this paper can be accessed here:

http://www.biomedcentral.com/1741-7015/9/48/prepub

## References

[B1] Reaven GM Banting lecture 1988. Role of insulin resistance in human disease Diabetes 1988 37 1595 1607 10.2337/diabetes.37.12.1595 3056758

[B2] Alberti KG Zimmet PZ Definition, diagnosis and classification of diabetes mellitus and its complications. Part 1: diagnosis and classification of diabetes mellitus provisional report of a WHO consultation Diabet Med 1998 15 539 553 10.1002/(SICI)1096-9136(199807)15:7<539::AID-DIA668>3.0.CO;2-S 9686693

[B3] Balkau B Charles MA Comment on the provisional report from the WHO consultation. European Group for the Study of Insulin Resistance (EGIR) Diabet Med 1999 16 442 443 10342346 10.1046/j.1464-5491.1999.00059.x

[B4] Executive Summary of The Third Report of The National Cholesterol Education Program (NCEP) Expert Panel on Detection, Evaluation, And Treatment of High Blood Cholesterol In Adults (Adult Treatment Panel III) JAMA 2001 285 2486 2497 10.1001/jama.285.19.2486 11368702

[B5] Alberti KG Zimmet P Shaw J The metabolic syndrome--a new worldwide definition Lancet 2005 366 1059 1062 10.1016/S0140-6736(05)67402-8 16182882

[B6] Grundy SM Brewer HB Jr Cleeman JI Smith SC Jr Lenfant C Definition of metabolic syndrome: report of the National Heart, Lung, and Blood Institute/American Heart Association conference on scientific issues related to definition Arterioscler Thromb Vasc Biol 2004 24 e13 e18 14766739 10.1161/01.ATV.0000111245.75752.C6

[B7] Alberti KG Eckel RH Grundy SM Zimmet PZ Cleeman JI Donato KA Fruchart JC James WP Loria CM Smith SC Jr International Diabetes Federation Task Force on Epidemiology and Prevention, National Heart, Lung, and Blood Institute, American Heart Association, World Heart Federation, International Atherosclerosis Society, International Association for the Study of Obesity Harmonizing the metabolic syndrome: a joint interim statement of the International Diabetes Federation Task Force on Epidemiology and Prevention; National Heart, Lung, and Blood Institute; American Heart Association; World Heart Federation; International Atherosclerosis Society; and International Association for the Study of Obesity Circulation 2009 120 1640 1645 10.1161/CIRCULATIONAHA.109.192644 19805654

[B8] Cameron AJ Shaw JE Zimmet PZ The metabolic syndrome: prevalence in worldwide populations Endocrinol Metab Clin North Am 2004 33 351 75 table 10.1016/j.ecl.2004.03.005 15158523

[B9] Cornier MA Dabelea D Hernandez TL Lindstrom RC Steig AJ Stob NR Van Pelt RE Wang H Eckel RH The metabolic syndrome Endocr Rev 2008 29 777 822 10.1210/er.2008-0024 18971485 PMC5393149

[B10] Hollman G Kristenson M The prevalence of the metabolic syndrome and its risk factors in a middle-aged Swedish population--mainly a function of overweight? Eur J Cardiovasc Nurs 2008 7 21 26 10.1016/j.ejcnurse.2007.05.003 17586094

[B11] Hillier TA Fagot-Campagna A Eschwege E Vol S Cailleau M Balkau B Weight change and changes in the metabolic syndrome as the French population moves towards overweight: the D.E.S.I.R. cohort Int J Epidemiol 2006 35 190 196 16373378 10.1093/ije/dyi281 PMC2062519

[B12] do Carmo I Dos Santos O Camolas J Vieira J Carreira M Medina L Reis L Myatt J Galvão-Teles A Overweight and obesity in Portugal: national prevalence in 2003-2005 Obes Rev 2008 9 11 19 18034792 10.1111/j.1467-789X.2007.00422.x

[B13] Ervin RB Prevalence of metabolic syndrome among adults 20 years of age and over, by sex, age, race and ethnicity, and body mass index: United States, 2003-2006 Natl Health Stat Report 2009 13 1 7 19634296

[B14] Ford ES Li C Zhao G Prevalence and correlates of metabolic syndrome based on a harmonious definition among adults in the US J Diabetes 2010 2 180 193 10.1111/j.1753-0407.2010.00078.x 20923483

[B15] Athyros VG Ganotakis ES Tziomalos K Papageorgiou AA Anagnostis P Griva T Kargiotis K Mitsiou EK Karagiannis A Mikhailidis DP Comparison of four definitions of the metabolic syndrome in a Greek (Mediterranean) population Curr Med Res Opin 2010 26 713 719 10.1185/03007991003590597 20078335

[B16] Harzallah F Alberti H Ben KF The metabolic syndrome in an Arab population: a first look at the new International Diabetes Federation criteria Diabet Med 2006 23 441 444 10.1111/j.1464-5491.2006.01866.x 16620275

[B17] Sharifi F Mousavinasab SN Saeini M Dinmohammadi M Prevalence of metabolic syndrome in an adult urban population of the west of Iran Exp Diabetes Res 2009 2009 136501 19893638 10.1155/2009/136501 PMC2773406

[B18] Timoteo A Santos R Lima S Mamede A Fernandes R Ferreira R Does the new International Diabetes Federation definition of metabolic syndrome improve prediction of coronary artery disease and carotid intima-media thickening? Rev Port Cardiol 2009 28 173 181 19438152

[B19] Ma WY Li HY Hung CS Lin MS Chiu FC Lin CH Shih SR Chuang LM Wei JN Metabolic syndrome defined by IDF and AHA/NHLBI correlates better to carotid intima-media thickness than that defined by NCEP ATP III and WHO Diabetes Res Clin Pract 2009 85 335 341 10.1016/j.diabres.2009.06.020 19608293

[B20] Zabetian A Hadaegh F Azizi F Prevalence of metabolic syndrome in Iranian adult population, concordance between the IDF with the ATPIII and the WHO definitions Diabetes Res Clin Pract 2007 77 251 257 10.1016/j.diabres.2006.12.001 17234299

[B21] Azizi F Salehi P Etemadi A Zahedi-Asl S Prevalence of metabolic syndrome in an urban population: Tehran Lipid and Glucose Study Diabetes Res Clin Pract 2003 61 29 37 10.1016/S0168-8227(03)00066-4 12849921

[B22] Fiuza M Cortez-Dias N Martins S Belo A Metabolic syndrome in Portugal: prevalence and implications for cardiovascular risk--results from the VALSIM Study Rev Port Cardiol 2008 27 1495 1529 19280993

[B23] Csaszar A Kekes E Abel T Papp R Kiss I Balogh S Prevalence of metabolic syndrome estimated by International Diabetes Federation criteria in a Hungarian population Blood Press 2006 15 101 106 10.1080/08037050600772284 16754273

[B24] Deepa M Farooq S Datta M Deepa R Mohan V Prevalence of metabolic syndrome using WHO, ATPIII and IDF definitions in Asian Indians: the Chennai Urban Rural Epidemiology Study (CURES-34) Diabetes Metab Res Rev 2007 23 127 134 10.1002/dmrr.658 16752431

[B25] Aguilar-Salinas CA Rojas R Gomez-Perez FJ Valles V Rios-Torres JM Franco A Olaiz G Rull JA Sepulveda J High prevalence of metabolic syndrome in Mexico Arch Med Res 2004 35 76 81 10.1016/j.arcmed.2003.06.006 15036804

[B26] Cameron AJ Magliano DJ Zimmet PZ Welborn T Shaw JE The metabolic syndrome in Australia: prevalence using four definitions Diabetes Res Clin Pract 2007 77 471 478 10.1016/j.diabres.2007.02.002 17350710

[B27] Misra A Khurana L Obesity and the metabolic syndrome in developing countries J Clin Endocrinol Metab 2008 93 S9 30 10.1210/jc.2008-1595 18987276

[B28] Diamanti-Kandarakis E Papavassiliou AG Kandarakis SA Chrousos GP Pathophysiology and types of dyslipidemia in PCOS Trends Endocrinol Metab 2007 18 280 285 10.1016/j.tem.2007.07.004 17692530

[B29] Giugliano D Ceriello A Esposito K The effects of diet on inflammation: emphasis on the metabolic syndrome J Am Coll Cardiol 2006 48 677 685 10.1016/j.jacc.2006.03.052 16904534

[B30] Reaven GM LeRoith D, Taylor S, Olefasky JM Insulin resistance and its consequences Diabetes Mellitus: A Fundamental and Clinical Text 2004 Philadelphia, PA: Lippincott, Williams 899 915

[B31] Zhu S Wang Z Heshka S Heo M Faith MS Heymsfield SB Waist circumference and obesity-associated risk factors among whites in the third National Health and Nutrition Examination Survey: clinical action thresholds Am J Clin Nutr 2002 76 743 749 12324286 10.1093/ajcn/76.4.743

[B32] Isomaa B Almgren P Tuomi T Forsén B Lahti K Nissén M Taskinen MR Groop L Cardiovascular morbidity and mortality associated with the metabolic syndrome Diabetes Care 2001 24 683 689 10.2337/diacare.24.4.683 11315831

[B33] Hu G Qiao Q Tuomilehto J Balkau B Borch-Johnsen K Pyorala K Prevalence of the metabolic syndrome and its relation to all-cause and cardiovascular mortality in nondiabetic European men and women Arch Intern Med 2004 164 1066 1076 10.1001/archinte.164.10.1066 15159263

[B34] Alexander CM Landsman PB Teutsch SM Haffner SM NCEP-defined metabolic syndrome, diabetes, and prevalence of coronary heart disease among NHANES III participants age 50 years and older Diabetes 2003 52 1210 1214 10.2337/diabetes.52.5.1210 12716754

[B35] Ford ES The metabolic syndrome and mortality from cardiovascular disease and all-causes: findings from the National Health and Nutrition Examination Survey II Mortality Study Atherosclerosis 2004 173 309 314 15064107 10.1016/j.atherosclerosis.2003.12.022

[B36] McNeill AM Rosamond WD Girman CJ Golden SH Schmidt MI East HE Ballantyne CM Heiss G The metabolic syndrome and 11-year risk of incident cardiovascular disease in the atherosclerosis risk in communities study Diabetes Care 2005 28 385 390 10.2337/diacare.28.2.385 15677797

[B37] Hunt KJ Resendez RG Williams K Haffner SM Stern MP National Cholesterol Education Program versus World Health Organization metabolic syndrome in relation to all-cause and cardiovascular mortality in the San Antonio Heart Study Circulation 2004 110 1251 1257 10.1161/01.CIR.0000140762.04598.F9 15326061

[B38] Lakka HM Laaksonen DE Lakka TA Niskanen LK Kumpusalo E Tuomilehto J Salonen JT The metabolic syndrome and total and cardiovascular disease mortality in middle-aged men JAMA 2002 288 2709 2716 10.1001/jama.288.21.2709 12460094

[B39] Ford ES Risks for all-cause mortality, cardiovascular disease, and diabetes associated with the metabolic syndrome: a summary of the evidence Diabetes Care 2005 28 1769 1778 10.2337/diacare.28.7.1769 15983333

[B40] Mottillo S Filion KB Genest J Joseph L Pilote L Poirier P Rinfret S Schiffrin EL Eisenberg MJ The metabolic syndrome and cardiovascular risk a systematic review and meta-analysis J Am Coll Cardiol 2010 56 1113 1132 10.1016/j.jacc.2010.05.034 20863953

[B41] Bruno G Merletti F Biggeri A Bargero G Ferrero S Runzo C Prina Cerai S Pagano G Cavallo-Perin P Casale Monferrato Study Metabolic syndrome as a predictor of all-cause and cardiovascular mortality in type 2 diabetes: the Casale Monferrato Study Diabetes Care 2004 27 2689 2694 10.2337/diacare.27.11.2689 15505006

[B42] Sattar N McConnachie A Shaper AG Blauw GJ Buckley BM de Craen AJ Ford I Forouhi NG Freeman DJ Jukema JW Lennon L Macfarlane PW Murphy MB Packard CJ Stott DJ Westendorp RG Whincup PH Shepherd J Wannamethee SG Can metabolic syndrome usefully predict cardiovascular disease and diabetes? Outcome data from two prospective studies Lancet 2008 371 1927 1935 10.1016/S0140-6736(08)60602-9 18501419

[B43] Gami AS Witt BJ Howard DE Erwin PJ Gami LA Somers VK Montori VM Metabolic syndrome and risk of incident cardiovascular events and death: a systematic review and meta-analysis of longitudinal studies J Am Coll Cardiol 2007 49 403 414 10.1016/j.jacc.2006.09.032 17258085

[B44] Mente A Yusuf S Islam S McQueen MJ Tanomsup S Onen CL Rangarajan S Gerstein HC Anand SS INTERHEART Investigators Metabolic syndrome and risk of acute myocardial infarction a case-control study of 26,903 subjects from 52 countries J Am Coll Cardiol 2010 55 2390 2398 10.1016/j.jacc.2009.12.053 20488312

[B45] Cabré JJ Martín F Costa B Piñol JL Llor JL Ortega Y Basora J Baldrich M Solà R Daniel J Hernández JM Saumell J Bladé J Sagarra R Basora T Montañés D Frigola JL Donado-Mazarrón A García-Vidal MT Sánchez-Oro I de Magriñà JM Urbaneja A Barrio F Vizcaíno J Sabaté JM Pascual I Revuelta V Metabolic syndrome as a cardiovascular disease risk factor: patients evaluated in primary care BMC Public Health 2008 8 251 10.1186/1471-2458-8-251 18647383 PMC2515316

[B46] Athyros VG Mikhailidis DP Papageorgiou AA Didangelos TP Ganotakis ES Symeonidis AN Daskalopoulou SS Kakafika AI Elisaf M METS-GREECE Collaborative Group Prevalence of atherosclerotic vascular disease among subjects with the metabolic syndrome with or without diabetes mellitus: the METS-GREECE Multicentre Study Curr Med Res Opin 2004 20 1691 1701 10.1185/030079904X5599 15587481

[B47] Teramura M Emoto M Araki T Yokoyama H Motoyama K Shinohara K Mori K Koyama H Shoji T Inaba M Nishizawa Y Clinical impact of metabolic syndrome by modified NCEP-ATPIII criteria on carotid atherosclerosis in Japanese adults J Atheroscler Thromb 2007 14 172 178 17704617 10.5551/jat.e505

[B48] Sattar N Gaw A Scherbakova O Ford I O'Reilly DS Haffner SM Isles C Macfarlane PW Packard CJ Cobbe SM Shepherd J Metabolic syndrome with and without C-reactive protein as a predictor of coronary heart disease and diabetes in the West of Scotland Coronary Prevention Study Circulation 2003 108 414 419 10.1161/01.CIR.0000080897.52664.94 12860911

[B49] Malik S Wong ND Franklin SS Kamath TV L'Italien GJ Pio JR Williams GR Impact of the metabolic syndrome on mortality from coronary heart disease, cardiovascular disease, and all causes in United States adults Circulation 2004 110 1245 1250 10.1161/01.CIR.0000140677.20606.0E 15326067

[B50] Iribarren C Go AS Husson G Sidney S Fair JM Quertermous T Hlatky MA Fortmann SP Metabolic syndrome and early-onset coronary artery disease: is the whole greater than its parts? J Am Coll Cardiol 2006 48 1800 1807 10.1016/j.jacc.2006.03.070 17084253

[B51] Nabipour I Amiri M Imami SR Jahfari SM Shafeiae E Nosrati A Iranpour D Soltanian AR The metabolic syndrome and nonfatal ischemic heart disease; a population-based study Int J Cardiol 2007 118 48 53 10.1016/j.ijcard.2006.06.017 16875744

[B52] Ginsberg HN MacCallum PR The obesity, metabolic syndrome, and type 2 diabetes mellitus pandemic: Part I. Increased cardiovascular disease risk and the importance of atherogenic dyslipidemia in persons with the metabolic syndrome and type 2 diabetes mellitus J Cardiometab Syndr 2009 4 113 119 10.1111/j.1559-4572.2008.00044.x 19614799 PMC2901596

[B53] Eckel RH Grundy SM Zimmet PZ The metabolic syndrome Lancet 2005 365 1415 1428 10.1016/S0140-6736(05)66378-7 15836891

[B54] Hanson RL Imperatore G Bennett PH Knowler WC Components of the "metabolic syndrome" and incidence of type 2 diabetes Diabetes 2002 51 3120 3127 10.2337/diabetes.51.10.3120 12351457

[B55] Grundy SM Cleeman JI Daniels SR Donato KA Eckel RH Franklin BA Gordon DJ Krauss RM Savage PJ Smith SC Jr Spertus JA Costa F American Heart Association; National Heart, Lung, and Blood Institute Diagnosis and management of the metabolic syndrome. An American Heart Association/National Heart, Lung, and Blood Institute Scientific Statement. Executive summary Cardiol Rev 2005 13 322 327 16708441

[B56] Ford ES Li C Sattar N Metabolic syndrome and incident diabetes: current state of the evidence Diabetes Care 2008 31 1898 1904 10.2337/dc08-0423 18591398 PMC2518368

[B57] Laaksonen DE Lakka HM Niskanen LK Kaplan GA Salonen JT Lakka TA Metabolic syndrome and development of diabetes mellitus: application and validation of recently suggested definitions of the metabolic syndrome in a prospective cohort study Am J Epidemiol 2002 156 1070 1077 10.1093/aje/kwf145 12446265

[B58] Hanley AJ Karter AJ Williams K Festa A D'Agostino RB Jr Wagenknecht LE Haffner SM Prediction of type 2 diabetes mellitus with alternative definitions of the metabolic syndrome: the Insulin Resistance Atherosclerosis Study Circulation 2005 112 3713 3721 10.1161/CIRCULATIONAHA.105.559633 16344402

[B59] Lorenzo C Okoloise M Williams K Stern MP Haffner SM The metabolic syndrome as predictor of type 2 diabetes: the San Antonio heart study Diabetes Care 2003 26 3153 3159 10.2337/diacare.26.11.3153 14578254

[B60] Cartier A Côté M Lemieux I Pérusse L Tremblay A Bouchard C Després JP Sex differences in inflammatory markers: what is the contribution of visceral adiposity? Am J Clin Nutr 2009 89 1307 1314 10.3945/ajcn.2008.27030 19297456

[B61] Onat A Ugur M Can G Yuksel H Hergenc G Visceral adipose tissue and body fat mass: predictive values for and role of gender in cardiometabolic risk among Turks Nutrition 2010 26 382 389 10.1016/j.nut.2009.05.019 19632090

[B62] Onat A Hergenc G Bulur S Ugur M Kucukdurmaz Z Can G The paradox of high apolipoprotein A-I levels independently predicting incident type-2 diabetes among Turks Int J Cardiol 2010 142 72 79 10.1016/j.ijcard.2008.12.066 19171400

[B63] Onat A Hergenc G Low-grade inflammation, and dysfunction of high-density lipoprotein and its apolipoproteins as a major driver of cardiometabolic risk Metabolism 2011 60 499 512 10.1016/j.metabol.2010.04.018 20580781

[B64] Onat A Can G Hergenc G Yazici M Karabulut A Albayrak S Serum apolipoprotein B predicts dyslipidemia, metabolic syndrome and, in women, hypertension and diabetes, independent of markers of central obesity and inflammation Int J Obes (Lond) 2007 31 1119 1125 10.1038/sj.ijo.0803552 17299378

[B65] Onat A Uyarel H Hergenç G Karabulut A Albayrak S Sari I Yazici M Keleş I Serum uric acid is a determinant of metabolic syndrome in a population-based study Am J Hypertens 2006 19 1055 1062 10.1016/j.amjhyper.2006.02.014 17027827

[B66] Onat A Hergenç G Karabulut A Türkmen S Doğan Y Uyarel H Can G Sansoy V Serum gamma glutamyltransferase as a marker of metabolic syndrome and coronary disease likelihood in nondiabetic middle-aged and elderly adults Prev Med 2006 43 136 139 10.1016/j.ypmed.2006.04.005 16714057

[B67] Onat A Ozhan H Erbilen E Albayrak S Küçükdurmaz Z Can G Keleş I Hergenç G Independent prediction of metabolic syndrome by plasma fibrinogen in men, and predictors of elevated levels Int J Cardiol 2009 135 211 217 10.1016/j.ijcard.2008.03.054 18582961

[B68] Ordovas JM Genetic links between diabetes mellitus and coronary atherosclerosis Curr Atheroscler Rep 2007 9 204 210 10.1007/s11883-007-0020-9 18241614

[B69] Stefan N Kantartzis K Machann J Schick F Thamer C Rittig K Balletshofer B Machicao F Fritsche A Häring HU Identification and characterization of metabolically benign obesity in humans Arch Intern Med 2008 168 1609 1616 10.1001/archinte.168.15.1609 18695074

[B70] Bravata DM Wells CK Concato J Kernan WN Brass LM Gulanski BI Two measures of insulin sensitivity provided similar information in a U.S. population J Clin Epidemiol 2004 57 1214 1217 10.1016/j.jclinepi.2004.05.001 15567640

[B71] St-Onge MP Janssen I Heymsfield SB Metabolic syndrome in normal-weight Americans: new definition of the metabolically obese, normal-weight individual Diabetes Care 2004 27 2222 2228 10.2337/diacare.27.9.2222 15333488

[B72] Matsuzawa Y The role of fat topology in the risk of disease Int J Obes (Lond) 2008 32 Suppl 7 S83 S92 10.1038/ijo.2008.243 19136997

[B73] Matsuzawa Y Adiponectin: a key player in obesity related disorders Curr Pharm Des 2010 16 1896 1901 10.2174/138161210791208893 20370675

[B74] Okamoto Y Kihara S Funahashi T Matsuzawa Y Libby P Adiponectin: a key adipocytokine in metabolic syndrome Clin Sci (Lond) 2006 110 267 278 10.1042/CS20050182 16464169

[B75] Kim JH Kim SH Im JA Lee DC The relationship between visfatin and metabolic syndrome in postmenopausal women Maturitas 2010 67 67 71 10.1016/j.maturitas.2010.05.002 20537826

[B76] S Standeven KF Hess K Carter AM Rice GI Cordell PA Balmforth AJ Lu B Scott DJ Turner AJ Hooper NM Grant PJ Neprilysin, obesity and the metabolic syndrome Int J Obes (Lond) in press 10.1038/ijo.2010.227 PMC3040694 21042321

[B77] Xu J Lloyd DJ Hale C Stanislaus S Chen M Sivits G Vonderfecht S Hecht R Li YS Lindberg RA Chen JL Jung DY Zhang Z Ko HJ Kim JK Véniant MM Fibroblast growth factor 21 reverses hepatic steatosis, increases energy expenditure, and improves insulin sensitivity in diet-induced obese mice Diabetes 2009 58 250 259 10.2337/db08-0392 18840786 PMC2606881

[B78] Stofkova A Resistin and visfatin: regulators of insulin sensitivity, inflammation and immunity Endocr Regul 2010 44 25 36 10.4149/endo_2010_01_25 20151765

[B79] Kahn SE Prigeon RL Schwartz RS Fujimoto WY Knopp RH Brunzell JD Porte D Jr Obesity, body fat distribution, insulin sensitivity and Islet beta-cell function as explanations for metabolic diversity J Nutr 2001 131 354S 360S 11160560 10.1093/jn/131.2.354S

[B80] Chrousos GP Gold PW The concepts of stress and stress system disorders. Overview of physical and behavioral homeostasis JAMA 1992 267 1244 1252 10.1001/jama.267.9.1244 1538563

[B81] Charmandari E Tsigos C Chrousos G Endocrinology of the stress response Annu Rev Physiol 2005 67 259 284 10.1146/annurev.physiol.67.040403.120816 15709959

[B82] Chrousos GP Kino T Glucocorticoid signaling in the cell. Expanding clinical implications to complex human behavioral and somatic disorders Ann N Y Acad Sci 2009 1179 153 166 10.1111/j.1749-6632.2009.04988.x 19906238 PMC2791367

[B83] Chrousos GP Stress and disorders of the stress system Nat Rev Endocrinol 2009 5 374 381 10.1038/nrendo.2009.106 19488073

[B84] Chrousos GP The role of stress and the hypothalamic-pituitary-adrenal axis in the pathogenesis of the metabolic syndrome: neuro-endocrine and target tissue-related causes Int J Obes Relat Metab Disord 2000 24 Suppl 2 S50 S55 10997609 10.1038/sj.ijo.0801278

[B85] Vgontzas AN Papanicolaou DA Bixler EO Hopper K Lotsikas A Lin HM Kales A Chrousos GP Sleep apnea and daytime sleepiness and fatigue: relation to visceral obesity, insulin resistance, and hypercytokinemia J Clin Endocrinol Metab 2000 85 1151 1158 10.1210/jc.85.3.1151 10720054

[B86] Stewart PM Boulton A Kumar S Clark PM Shackleton CH Cortisol metabolism in human obesity: impaired cortisone→cortisol conversion in subjects with central adiposity J Clin Endocrinol Metab 1999 84 1022 1027 10.1210/jc.84.3.1022 10084590

[B87] Valsamakis G Anwar A Tomlinson JW Shackleton CH McTernan PG Chetty R Wood PJ Banerjee AK Holder G Barnett AH Stewart PM Kumar S 11beta-hydroxysteroid dehydrogenase type 1 activity in lean and obese males with type 2 diabetes mellitus J Clin Endocrinol Metab 2004 89 4755 4761 10.1210/jc.2003-032240 15356090

[B88] Gathercole LL Stewart PM Targeting the pre-receptor metabolism of cortisol as a novel therapy in obesity and diabetes J Steroid Biochem Mol Biol 2010 122 21 27 10.1016/j.jsbmb.2010.03.060 20347978

[B89] Vgontzas AN Chrousos GP Sleep-disordered breathing, sleepiness, and insulin resistance: is the latter a consequence, a pathogenetic factor, or both? Sleep Med 2002 3 389 391 10.1016/S1389-9457(02)00067-9 14592169

[B90] Vgontzas AN Chrousos GP Sleep, the hypothalamic-pituitary-adrenal axis, and cytokines: multiple interactions and disturbances in sleep disorders Endocrinol Metab Clin North Am 2002 31 15 36 10.1016/S0889-8529(01)00005-6 12055986

[B91] Vgontzas AN Bixler EO Chrousos GP Obesity-related sleepiness and fatigue: the role of the stress system and cytokines Ann N Y Acad Sci 2006 1083 329 344 10.1196/annals.1367.023 17148748

[B92] Vgontzas AN Bixler EO Chrousos GP Sleep apnea is a manifestation of the metabolic syndrome Sleep Med Rev 2005 9 211 224 10.1016/j.smrv.2005.01.006 15893251

[B93] Nader N Chrousos GP Kino T Interactions of the circadian CLOCK system and the HPA axis Trends Endocrinol Metab 2010 21 277 286 10.1016/j.tem.2009.12.011 20106676 PMC2862789

[B94] Furukawa S Fujita T Shimabukuro M Iwaki M Yamada Y Nakajima Y Nakayama O Makishima M Matsuda M Shimomura I Increased oxidative stress in obesity and its impact on metabolic syndrome J Clin Invest 2004 114 1752 1761 15599400 10.1172/JCI21625 PMC535065

[B95] Grattagliano I Vendemiale G Boscia F Micelli-Ferrari T Cardia L Altomare E Oxidative retinal products and ocular damages in diabetic patients Free Radic Biol Med 1998 25 369 372 10.1016/S0891-5849(98)00059-8 9680184

[B96] Wei Y Sowers JR Nistala R Gong H Uptergrove GM Clark SE Morris EM Szary N Manrique C Stump CS Angiotensin II-induced NADPH oxidase activation impairs insulin signaling in skeletal muscle cells J Biol Chem 2006 281 35137 35146 10.1074/jbc.M601320200 16982630

[B97] Blendea MC Jacobs D Stump CS McFarlane SI Ogrin C Bahtyiar G Stas S Kumar P Sha Q Ferrario CM Sowers JR Abrogation of oxidative stress improves insulin sensitivity in the Ren-2 rat model of tissue angiotensin II overexpression Am J Physiol Endocrinol Metab 2005 288 E353 E359 10.1152/ajpendo.00402.2004 15494608

[B98] Giacchetti G Sechi LA Rilli S Carey RM The renin-angiotensin-aldosterone system, glucose metabolism and diabetes Trends Endocrinol Metab 2005 16 120 126 10.1016/j.tem.2005.02.003 15808810

[B99] Nabeshima Y Tazuma S Kanno K Hyogo H Chayama K Deletion of angiotensin II type I receptor reduces hepatic steatosis J Hepatol 2009 50 1226 1235 10.1016/j.jhep.2009.01.018 19395110

[B100] Cooper SA Whaley-Connell A Habibi J Wei Y Lastra G Manrique C Stas S Sowers JR Renin-angiotensin-aldosterone system and oxidative stress in cardiovascular insulin resistance Am J Physiol Heart Circ Physiol 2007 293 H2009 H2023 10.1152/ajpheart.00522.2007 17586614

[B101] Williams JS Williams GH 50th anniversary of aldosterone J Clin Endocrinol Metab 2003 88 2364 2372 10.1210/jc.2003-030490 12788829

[B102] Tirosh A Garg R Adler GK Mineralocorticoid receptor antagonists and the metabolic syndrome Curr Hypertens Rep 2010 12 252 257 10.1007/s11906-010-0126-2 20563672 PMC2948675

[B103] Sowers JR Whaley-Connell A Epstein M Narrative review: the emerging clinical implications of the role of aldosterone in the metabolic syndrome and resistant hypertension Ann Intern Med 2009 150 776 783 19487712 10.7326/0003-4819-150-11-200906020-00005 PMC2824330

[B104] Schiffrin EL Effects of aldosterone on the vasculature Hypertension 2006 47 312 318 10.1161/01.HYP.0000201443.63240.a7 16432039

[B105] Brown NJ Aldosterone and vascular inflammation Hypertension 2008 51 161 167 10.1161/HYPERTENSIONAHA.107.095489 18172061

[B106] Krutzfeldt J Stoffel M MicroRNAs: a new class of regulatory genes affecting metabolism Cell Metab 2006 4 9 12 10.1016/j.cmet.2006.05.009 16814728

[B107] Jackson RJ Standart N How do microRNAs regulate gene expression? Sci STKE 2007 2007 re1 10.1126/stke.3672007re1 17200520

[B108] Heneghan HM Miller N Kerin MJ Role of microRNAs in obesity and the metabolic syndrome Obes Rev 2010 11 354 361 10.1111/j.1467-789X.2009.00659.x 19793375

[B109] Krützfeldt J Rajewsky N Braich R Rajeev KG Tuschl T Manoharan M Stoffel M Silencing of microRNAs in vivo with 'antagomirs' Nature 2005 438 685 689 10.1038/nature04303 16258535

[B110] Ravelli GP Stein ZA Susser MW Obesity in young men after famine exposure in utero and early infancy N Engl J Med 1976 295 349 353 10.1056/NEJM197608122950701 934222

[B111] Kensara OA Wootton SA Phillips DI Patel M Jackson AA Elia M Fetal programming of body composition: relation between birth weight and body composition measured with dual-energy X-ray absorptiometry and anthropometric methods in older Englishmen Am J Clin Nutr 2005 82 980 987 16280428 10.1093/ajcn/82.5.980

[B112] Gale CR Martyn CN Kellingray S Eastell R Cooper C Intrauterine programming of adult body composition J Clin Endocrinol Metab 2001 86 267 272 10.1210/jc.86.1.267 11232011

[B113] Simmons RA Templeton LJ Gertz SJ Intrauterine growth retardation leads to the development of type 2 diabetes in the rat Diabetes 2001 50 2279 2286 10.2337/diabetes.50.10.2279 11574409

[B114] Germani D Puglianiello A Cianfarani S Uteroplacental insufficiency down regulates insulin receptor and affects expression of key enzymes of long-chain fatty acid (LCFA) metabolism in skeletal muscle at birth Cardiovasc Diabetol 2008 7 14 10.1186/1475-2840-7-14 18485240 PMC2396605

[B115] Xita N Tsatsoulis A Fetal origins of the metabolic syndrome Ann N Y Acad Sci 2010 1205 148 155 10.1111/j.1749-6632.2010.05658.x 20840267

[B116] Fernandez-Twinn DS Ozanne SE Early life nutrition and metabolic programming Ann N Y Acad Sci 2010 1212 78 96 10.1111/j.1749-6632.2010.05798.x 21070247

[B117] Sakka SD Loutradis D Kanaka-Gantenbein C Margeli A Papastamataki M Papassotiriou I Chrousos GP Absence of insulin resistance and low-grade inflammation despite early metabolic syndrome manifestations in children born after in vitro fertilization Fertil Steril 2010 94 1693 1699 10.1016/j.fertnstert.2009.09.049 20045517

[B118] Ceelen M van Weissenbruch MM Vermeiden JP van Leeuwen FE Delemarre-van de Waal HA Cardiometabolic differences in children born after in vitro fertilization: follow-up study J Clin Endocrinol Metab 2008 93 1682 1688 10.1210/jc.2007-2432 18285409

[B119] Miles HL Hofman PL Peek J Harris M Wilson D Robinson EM Gluckman PD Cutfield WS In vitro fertilization improves childhood growth and metabolism J Clin Endocrinol Metab 2007 92 3441 3445 10.1210/jc.2006-2465 17566097

[B120] Weiss R Dziura J Burgert TS Tamborlane WV Taksali SE Yeckel CW Allen K Lopes M Savoye M Morrison J Sherwin RS Caprio S Obesity and the metabolic syndrome in children and adolescents N Engl J Med 2004 350 2362 2374 10.1056/NEJMoa031049 15175438

[B121] Hannon TS Rao G Arslanian SA Childhood obesity and type 2 diabetes mellitus Pediatrics 2005 116 473 480 10.1542/peds.2004-2536 16061606

[B122] Chen W Srinivasan SR Elkasabany A Berenson GS Cardiovascular risk factors clustering features of insulin resistance syndrome (Syndrome X) in a biracial (Black-White) population of children, adolescents, and young adults: the Bogalusa Heart Study Am J Epidemiol 1999 150 667 674 10512420 10.1093/oxfordjournals.aje.a010069

[B123] Cook S Weitzman M Auinger P Nguyen M Dietz WH Prevalence of a metabolic syndrome phenotype in adolescents: findings from the third National Health and Nutrition Examination Survey, 1988-1994 Arch Pediatr Adolesc Med 2003 157 821 827 10.1001/archpedi.157.8.821 12912790

[B124] Chen W Bao W Begum S Elkasabany A Srinivasan SR Berenson GS Age-related patterns of the clustering of cardiovascular risk variables of syndrome X from childhood to young adulthood in a population made up of black and white subjects: the Bogalusa Heart Study Diabetes 2000 49 1042 1048 10.2337/diabetes.49.6.1042 10866058

[B125] Csabi G Torok K Jeges S Molnar D Presence of metabolic cardiovascular syndrome in obese children Eur J Pediatr 2000 159 91 94 10.1007/PL00013812 10653338

[B126] Moreno LA Pineda I Rodriguez G Fleta J Sarria A Bueno M Waist circumference for the screening of the metabolic syndrome in children Acta Paediatr 2002 91 1307 1312 10.1111/j.1651-2227.2002.tb02825.x 12578286

[B127] Duncan GE Li SM Zhou XH Prevalence and trends of a metabolic syndrome phenotype among U.A. adolescents, 1999-2000 Diabetes Care 2004 27 2438 2443 10.2337/diacare.27.10.2438 15451913

[B128] Cruz ML Weigensberg MJ Huang TT Ball G Shaibi GQ Goran MI The metabolic syndrome in overweight Hispanic youth and the role of insulin sensitivity J Clin Endocrinol Metab 2004 89 108 113 10.1210/jc.2003-031188 14715836

[B129] de Ferranti SD Gauvreau K Ludwig DS Neufeld EJ Newburger JW Rifai N Prevalence of the metabolic syndrome in American adolescents: findings from the Third National Health and Nutrition Examination Survey Circulation 2004 110 2494 2497 10.1161/01.CIR.0000145117.40114.C7 15477412

[B130] Lambert M Paradis G O'Loughlin J Delvin EE Hanley JA Levy E Insulin resistance syndrome in a representative sample of children and adolescents from Quebec, Canada Int J Obes Relat Metab Disord 2004 28 833 841 10.1038/sj.ijo.0802694 15170466

[B131] Goodman E Dolan LM Morrison JA Daniels SR Factor analysis of clustered cardiovascular risks in adolescence: obesity is the predominant correlate of risk among youth Circulation 2005 111 1970 1977 10.1161/01.CIR.0000161957.34198.2B 15837951

[B132] Pervanidou P Kanaka-Gantenbein C Chrousos GP Assessment of metabolic profile in a clinical setting Curr Opin Clin Nutr Metab Care 2006 9 589 595 10.1097/01.mco.0000241669.24923.8d 16912555

[B133] Brambilla P Lissau I Flodmark CE Moreno LA Widhalm K Wabitsch M Pietrobelli A Metabolic risk-factor clustering estimation in children: to draw a line across pediatric metabolic syndrome Int J Obes (Lond) 2007 31 591 600 10.1038/sj.ijo.0803581 17384660

[B134] Zimmet P Alberti KG Kaufman F Tajima N Silink M Arslanian S Wong G Bennett P Shaw J Caprio S IDF Consensus Group The metabolic syndrome in children and adolescents - an IDF consensus report Pediatr Diabetes 2007 8 299 306 10.1111/j.1399-5448.2007.00271.x 17850473

[B135] Goran MI Gower BA Longitudinal study on pubertal insulin resistance Diabetes 2001 50 2444 2450 10.2337/diabetes.50.11.2444 11679420

[B136] Viner RM Segal TY Lichtarowicz-Krynska E Hindmarsh P Prevalence of the insulin resistance syndrome in obesity Arch Dis Child 2005 90 10 14 10.1136/adc.2003.036467 15613503 PMC1720077

[B137] Pilia S Casini MR Foschini ML Minerba L Musiu MC Marras V Civolani P Loche S The effect of puberty on insulin resistance in obese children J Endocrinol Invest 2009 32 401 405 19794287 10.1007/BF03346475

[B138] Goodman E Daniels SR Meigs JB Dolan LM Instability in the diagnosis of metabolic syndrome in adolescents Circulation 2007 115 2316 2322 10.1161/CIRCULATIONAHA.106.669994 17420347 PMC2626638

[B139] Goodman E Li C Tu YK Ford E Sun SS Huang TT Stability of the factor structure of the metabolic syndrome across pubertal development: confirmatory factor analyses of three alternative models J Pediatr 2009 155 S5 S8 19732562 10.1016/j.jpeds.2009.04.045 PMC3763727

[B140] Pervanidou P Kanaka-Gantenbein C Lazopoulou N Karamouzis I Bastaki D Julius A Chrousos GP Inconsistency of Metabolic Syndrome Diagnosis and Correlation of Morning Serum Cortisol Concentrations with Stability of Metabolic Abnormalities in Obese Children and Adolescents Hormone Research 2009 72 Suppl 3 283

[B141] Magnussen CG Koskinen J Chen W Thomson R Schmidt MD Srinivasan SR Kivimäki M Mattsson N Kähönen M Laitinen T Taittonen L Rönnemaa T Viikari JS Berenson GS Juonala M Raitakari OT Pediatric metabolic syndrome predicts adulthood metabolic syndrome, subclinical atherosclerosis, and type 2 diabetes mellitus but is no better than body mass index alone: the Bogalusa Heart Study and the Cardiovascular Risk in Young Finns Study Circulation 2010 122 1604 1611 10.1161/CIRCULATIONAHA.110.940809 20921439 PMC3388503

[B142] Einhorn D Reaven GM Cobin RH Ford E Ganda OP Handelsman Y Hellman R Jellinger PS Kendall D Krauss RM Neufeld ND Petak SM Rodbard HW Seibel JA Smith DA Wilson PW American College of Endocrinology position statement on the insulin resistance syndrome Endocr Pract 2003 9 237 252 12924350

[B143] Ford ES Giles WH Dietz WH Prevalence of the metabolic syndrome among US adults: findings from the third National Health and Nutrition Examination Survey JAMA 2002 287 356 359 10.1001/jama.287.3.356 11790215

[B144] Ford ES Giles WH A comparison of the prevalence of the metabolic syndrome using two proposed definitions Diabetes Care 2003 26 575 581 10.2337/diacare.26.3.575 12610004

[B145] Park YW Zhu S Palaniappan L Heshka S Carnethon MR Heymsfield SB The metabolic syndrome: prevalence and associated risk factor findings in the US population from the Third National Health and Nutrition Examination Survey, 1988-1994 Arch Intern Med 2003 163 427 436 10.1001/archinte.163.4.427 12588201 PMC3146257

